# Engaging Community Health Workers to Enhance Modern Contraceptive Uptake Among Young First-Time Parents in Five Cities of Uttar Pradesh

**DOI:** 10.9745/GHSP-D-22-00170

**Published:** 2024-05-21

**Authors:** Mukesh Kumar Sharma, Emily Das, Hitesh Sahni, Jessica Mirano, Kate Graham, Abhishek Kumar, Clea Finkle

**Affiliations:** a Population Services International, New Delhi, India.; b William H. Gates Sr. Institute for Population and Reproductive Health, Johns Hopkins Bloomberg School of Public Health, Baltimore, MD, USA.; c Population Council Consulting Pvt. Ltd., New Delhi, India.; d Independent consultant, Seattle, WA, USA.

## Abstract

**Introduction:**

Young newly married women and first-time parents (FTPs), particularly those living in slum settlements, have a high unmet need for modern contraceptive methods to limit and space births. We describe an intervention in which adolescents and youth sexual and reproductive health (AYSRH) services tailored to FTPs were incorporated into the government’s existing family planning (FP) program in 5 cities of Uttar Pradesh. We examined the effect of this intervention on modern contraceptive use among FTPs aged 15–24 years.

**Methods:**

To assess the effect of this pilot, in 2019, 1 year after the implementation of the program, we analyzed community-based output tracking survey data on 549 married women who are FTPs in the pilot cities. These FTPs were compared with 253 women who were FTPs from other cities where the program was implemented without a specific focus on FTPs. Descriptive statistics and multivariate logistic regression analysis were applied to understand the association between exposure to FP information, either through accredited social health activists or through service delivery points, and use of modern contraceptives.

**Results:**

Use of modern contraceptives was higher among FTPs in the 5 pilot cities than non-pilot cities (39% vs. 32%; *P*<.05). The interaction effect of city type and exposure to the information showed a positive association between modern contraceptive use and program exposure, greater in pilot cities than non-pilot cities.

**Conclusions:**

Higher uptake of modern contraceptives among young women may be achieved when an FTP-focused intervention is layered on the government’s existing FP programs. Future studies with a longer duration of implementation, in a wider geography, and with longitudinal design are recommended to provide more robust measures of high impact intervention/practices in urban areas.

## Abstract (precis)

Adding tailored information and counseling on contraceptive methods for young married and first-time parents to an existing family planning program can enhance the use of modern contraceptive methods for this population.

## Teaser

**Key Findings:**

- In Uttar Pradesh, India, young married first-time parents (FTPs) have a higher unmet need for family planning (FP) compared to married women in general, and their modern contraceptive prevalence rate is low.
- After accredited social health activists were coached and mentored in providing information and counseling on FP and healthy birth spacing with a particular focus on FTPs, the exposure to FP information was documented in 5 cities where the intervention was piloted.
- There was a positive association between any FTP intervention exposure and modern contraceptive method use, and this association is relatively stronger in cities where an intentional focus on FTPs was added to a functional family planning program than in cities without FTP interventions.

**Key Implications:**

- Program managers should consider tailoring specific FP programs by engaging community health workers and improving outreach services to ensure young FTPs have access to a range of contraceptive methods.
- Interventions that support FP counseling and methods, specifically for young FTPs, are critical for achieving goals outlined in Family Planning 2030 and the related Sustainable Development Goals.

## INTRODUCTION

In India, an estimated 1.5 million girls marry each year before reaching age 18 years,^1^ and childbearing almost always takes place within the marital union among young women aged younger than 25 years.^2^^–^^4^ The most recent estimates by the National Family and Health Survey indicate that 23% of women aged 20–24 years were married before age 18 years, and 7% of young women (15–19 years) had either already become mothers or were pregnant for the first time.^5^ This proportion was higher in the state of Uttar Pradesh, which has an estimated 22.3 million women aged 15–24 years,^6^ with only 26% of them using modern methods of contraception to space births.^5^ Early marriage among young women results in early and repeated childbearing over their lifetimes, which not only deprives them of educational and economic opportunities but also increases health risks for both the women and their newborns.^1^^,^^7^^,^^8^

Traditionally, the family planning (FP) and reproductive health programs of the Government of India have focused on married women (in general, not age specific), although in recent years, they have expanded their scope to include unmarried adolescents. The programs have inadvertently neglected married young first-time parents (FTPs) despite this group’s enormous unmet need for FP, which is 21.1% among FTPs aged 15–24 years compared to 9.4% among all married women aged 15–49 years.^9^ Young newly married women and FTPs are pressured by gendered social norms to have early and closely spaced, repeat births to prove their fertility and value.^10^ The vulnerabilities and social exclusion of FTPs are further heightened among the urban poor living in slum settlements. The urban poor often have high fertility, low use of health services, poor maternal outcomes, and low contraceptive use.^11^ FTPs in poor urban settings often face barriers to accessing quality reproductive health services because they are either overlooked by policies or are not reached by programs.^12^

The FP programs in India have inadvertently neglected married young first-time parents, despite this population having significant unmet need.

Since the launch of India’s official FP program in 1952, its focus has been almost exclusively on permanent methods, such as male and female sterilization, with an overarching aim of controlling population growth. Subsequently, reversible methods, such as intrauterine devices (IUDs), condoms, and oral contraceptive pills, were included in the official FP program, but their use has remained low. More recently, the Government of India included the injectable contraceptive medroxyprogesterone acetate (brand name Antara) and a weekly contraceptive pill (Chhaya; earlier marketed as Saheli) under India’s National Family Planning Program. Despite these changes in policies and the availability of a wider choice of methods, the proportion of women using a modern spacing method, such as condoms, in the state of Uttar Pradesh was only 27.5% in 2019–2021,^5^ whereas female sterilization has slightly increased from 36% in 2015–2016 to 38% in 2019–2021.^5^

In India, community health workers, known as accredited social health activists (ASHAs), are typically responsible for in-person counseling on FP^13^ and for the distribution of condoms, oral contraceptive pills, and emergency contraceptive pills. ASHAs are often inclined to encourage women to opt for female sterilization over other methods because of the immediate monetary rewards related to the method.^14^^,^^15^ Again, ASHAs rarely promote the ensuring spacing at birth (ESB) scheme as they have to wait for 2 years after a woman adopts a spacing method to receive the related incentives. This financing scheme constitutes a barrier for ASHAs to promote other contraceptive methods.^14^ Consequently, in India, uptake and use of male sterilization (0.3%), IUDs/postpartum IUDs (2.1%), injectables (0.6%), and pills (5.1%) continue to be very low.^5^

Furthermore, recent data show that in urban Uttar Pradesh, women adopt female sterilization only after having an average of 3.5 children.^5^ Hence, the unmet need for spacing pregnancies is higher (9%) than the unmet need for limiting (4%) among women who are FTPs.^5^ Therefore, if given the opportunity, FTPs are likely to adopt short-term/reversible methods rather than permanent methods, which, heretofore, have been the most highly promoted.

Despite the challenges, evidence suggests that ASHA-level interventions in low-income settings in India can be effective in improving maternal and child health services, including FP.^14^^,^^15^ However, there is no such scientific evidence available in urban poor settings of India to demonstrate the effectiveness of such interventions focusing on FTPs aged younger than 25 years.

In this article, we describe a government-led, potentially cost-effective, and scalable service delivery model to address unmet need for modern contraceptives among young FTPs and analyze whether the intervention has any association with contraceptive uptake among young FTPs in pilot cities compared to those in non-pilot cities. The findings from this study can inform prioritization and modifications of current FP policies and other structures related to improving reach to FTPs and the quality of FP services provided by community health workers to FTPs aged 15–24 years.

## PROGRAM DESCRIPTION

In October 2017, The Challenge Initiative for Healthy Cities (TCIHC) in India, led by Population Services International India in partnership with the National Health Mission and with financial support from the William H. Gates Sr. Institute for Population and Reproductive Health, initiated its support for FP services across 20 self-selected cities in Uttar Pradesh and continued until June 2020. Self-selection of cities was based on the expression of interest by cities to incorporate adolescents and youth sexual and reproductive health (AYSRH) services with government-leveraged resources. In August 2018, TCIHC incorporated new AYSRH interventions into the Government of India’s urban health service delivery model in 5 pilot cities of the 20 self-selected cities of Uttar Pradesh: Allahabad, Firozabad, Gorakhpur, Saharanpur, and Varanasi. The initiative supported the National Health Mission to deliver reproductive health services, including long- and short-acting reversible contraceptive methods, to young married women aged younger than 25 years by strengthening the capacity of urban local governments, health providers at urban primary health centers, community health workers, such as ASHAs, and advocacy with local government on releasing incentives for ESB schemes.^16^

The initiative sought the active involvement of local governments to strategically draw their attention to FTPs aged younger than 25 years because the unmet need for FP is high among this group, which has the lowest modern contraceptive prevalence rate (mCPR) in Uttar Pradesh. The government “layered” AYSRH activities onto the existing reproductive health service delivery mechanisms in these 5 pilot cities. As the first step for layering AYSRH activities in 2018, a total of 994 ASHAs in 5 pilot cities were trained and oriented on FP with particular focus on FTPs. Further, TCIHC coaches, namely field program associates, used the ASHA-auxiliary nurse midwife monthly meeting platform at facilities to coach and mentor ASHAs over a period of 6 months. Six sessions were conducted for ASHAs on topics on listing and mapping urban slums; updating the urban health index register (UHIR), a comprehensive register maintained by an ASHA for recording health care services provided within her catchment area, identifying FTPs from the UHIR; and reaching and counseling FTPs on healthy spacing and connecting with a service location, as well as on the benefits of the ESB scheme and follow-up for retention of users and continuation of methods.

FTPs aged younger than 25 years have a high unmet need for FP and the lowest modern contraceptive prevalence rate in Uttar Pradesh.

TCIHC supported the government to publicize the ESB scheme and, through meetings, coaching, training, and pamphlets, positioned the ESB as an ASHA Investment Plan. ASHAs began to consider UHIR as a long-term investment plan that could motivate them to save for the future when they could easily retrieve information to claim their rightful incentives for promoting spacing methods. This encouraged ASHAs to channel their efforts to promote spacing methods among FTPs and avail the ESB scheme. Spacing requires access to short-term methods available in the government basket of choices, and this is desired by FTPs.

At the facility level, TCIHC conducted whole-site orientations—a high-impact intervention that ensures all facility staff receive training to gain a basic understanding of FP/AYSRH and its benefits—and supported health staff from urban areas to establish adolescent- and youth-friendly health services to overcome provider bias to ensure providers and facility staff were aware of the latest medical guidelines and had accurate knowledge on all the methods available for young FTPs.

The detailed package of interventions in the pilot versus non-pilot cities is described in Table 1. More details about the program are provided in the Supplement.

**TABLE 1.** Package of Interventions Implemented in the AYSRH Program in Pilot and Non-Pilot Cities

**Interventions**	**Pilot Cities**	**Non-Pilot Cities**
Dedicated and focused day for FP services for all married women aged 15–49 years	Yes	Yes
Dedicated and focused for FP services for FTPs aged 15–24 years	Yes	No
Coached ASHAs to update UHIRs to make FP data visible for all aged women 15–49 years	Yes	Yes
Coached ASHAs to update UHIRs to make FP data visible for FTPs aged 15–24 years and recorded for prioritization	Yes	No
Supported the ASHAs to understand the ensuring spacing at birth scheme and to help FTPs choose a method of their choice	Yes	No
Established adolescent-friendly health services	Yes	No
Trained at least 1 auxiliary nurse midwife/staff nurse at each urban primary health center in adolescent- and youth-friendly services	Yes	
Trained staff on adolescent- and youth-friendly counseling skills related to FP and contraception	Yes	No^1^
Whole-site orientation for urban primary health center staff on AYSRH and FTPs	Yes	No^2^

Abbreviations: ASHA, accredited social health activist; AYSRH, adolescent and youth sexual and reproductive health; FP, family planning; FTP, first-time parent; UHIR, urban health index register.

^1^ No specific focus on adolescents and young people, but general training on FP.

^2^ No specific focus on adolescents and young people, but general whole-site orientation.

## METHODS

### Data

We analyzed data from an output tracking survey (OTS) conducted in September 2019, 1 year after the implementation of the program. The survey was conducted among married women aged 15–49 years in 14 selected cities of Uttar Pradesh, Madhya Pradesh, and Odisha. The survey collected information on knowledge about contraceptive methods, contraceptive use, method mix, demand for FP services, and exposure to FP information through various community and health facility-based platforms.

To select currently married women aged 15–49 years, the OTS adopted a multistage stratified random sampling design: (1) slum and non-slum wards in pilot and non-pilot cities were selected and demarcated under the TCIHC program; (2) within the slum and non-slum areas, 15 slum lots (an area with an average of 300 households) and 15 census enumeration blocks with an average of 300 households, respectively, were randomly selected; (3) 20 households were selected from each lot using the systematic random sampling strategy in which a random household from the lots was selected and the next household was selected following the interval as calculated after dividing the total number of households in a lot by the required number of households in that particular lot until 20 households were selected; (4) from each of the 20 selected households, 1 married woman of reproductive age was selected for the interview. The total sample size of the OTS was 8,319 currently married women aged 15–49 years.

In the 5 pilot cities of Uttar Pradesh, 1 eligible woman aged 15–24 years from each of the selected households in slum areas was interviewed, resulting in complete interviews of 549 FTPs aged 15–24 years (women who have only 1 living child, this also includes women aged 15–24 years who were pregnant at the time of the survey). A comparison group of FTPs was drawn from the OTS data from the non-pilot cities after excluding the responses from women aged 25–49 years. The FTPs in non-pilot cities were living in slum areas and did not receive any FTP-specific intervention but were exposed to the government-led general FP programs. Hence, we were able to derive comparable estimates to those from pilot cities. Participation of all respondents was voluntary and confidential. Participants gave their informed verbal consent before starting the interview.

### Outcome Variable

The main outcome variable was the use of modern contraceptive methods, including female sterilization, male sterilization, IUDs, injectables, oral contraceptive pills (daily, weekly pill/Chaaya), emergency contraceptive pills, male condoms, standard days method, lactational amenorrhea method, and others. All first-time young mothers aged 15–24 years who reported that they were using any of the modern methods at the time of the survey were considered to be modern contraceptive users, whereas those using traditional methods (such as rhythm method or withdrawal) or not using any contraceptive method were considered to be a non-user of modern contraceptives.

### Key Predictors: Measures of Family Planning Information Exposure

The key predictor variables used in the study were FTP’s exposure to FP information, which was defined as:

- Exposure to FP information through ASHAs: If a first-time young mother met an ASHA for FP-related information or received counseling on FP in the 6 months before the survey date.
- Exposure to FP information at a service delivery point (SDP): If a first-time young mother visited an SDP, such as an urban primary health center, outreach camp, or urban health and nutrition day, to seek FP-related information or counseling or services in the 6 months before the survey date. While considering the SDPs, we did not include private health facilities, as the entire program was focused on public health system.
- Any exposure either through ASHAs or through SDP: A combination of the afore-mentioned exposures and defined as if a first-time young mother met an ASHA or visited any of the SDPs for FP-related information or counseling or services in the 6 months before the survey date.

### Confounders

Although the main focus of the study was to examine the association between the key predictors and modern method use among FTPs, the analyses were also adjusted for background characteristics of FTPs, including age of women (18–21 years, 22–24 years), marital duration (less than 5 years, 5 years or more), completed years of schooling (none, less than 6 years, 6–9 years, 12 years or more), current working status (yes, no), religion (Hindu, non-Hindu), caste (scheduled caste/scheduled tribes, other classes); household wealth tertile (poor, middle, rich). These background characteristics were found to be associated with knowledge and access to contraceptives and hence influence overall modern method use.^17^^–^^21^ These studies documented that the background characteristics influence through knowledge level of method, knowledge of source of methods, and accessibility of contraceptive methods, which all influence the overall use of modern methods. Hence, if we do not account for these background variables, our results may be biased to a certain extent. Therefore, we adjusted these variables in the regression analysis by putting them in the model along with the key predictors (previously mentioned). Household wealth tertile was computed using variables on household amenities, consumer durables, household goods, assets, and size of land holding by applying principal component analysis.^22^^–^^24^

### Statistical Analysis

We conducted univariate and bivariate analyses to examine the prevalence of exposure to FP information among FTPs between pilot and non-pilot cities. We also used bivariate analysis to examine the prevalence of modern contraceptive use between pilot and non-pilot cities, between information exposure and no exposure, and across selected background characteristics of women. The bivariate analyses were substantiated with a z-test to examine significant differences in outcome variables between the groups/subgroups. We used multivariate binary logistic regression to examine the association between information exposure and modern contraceptive use in pilot and non-pilot cities. Binary logistic regression analysis was applied because the outcome variable was binary (1=use of the modern method, 0=otherwise). In the multivariate analysis, we estimated the interaction effect of city type (pilot and non-pilot) and information exposure (exposure on FP and no exposure). We did this to examine the association between information exposure and contraceptive use in the pilot cities compared to no information exposure in non-pilot cities. We adopted a total of 3 models by creating 3 types of interaction variables—interaction between city type and exposure to FP information through ASHAs, interaction with city type and exposure to FP information through SDPs, and interaction between city type and exposure to FP information through ASHAs or/and SDPs. The regression analyses in each of the models were adjusted for the selected sociodemographic confounders. Results obtained from the analyses are presented in terms of odds ratios (OR) along with 95% confidence intervals (95% CI). An OR greater than 1 indicates higher odds of modern method use. All the analyses were conducted in Stata version 14.0.

### Ethical Approval

Before the survey was conducted, approvals were granted by the Institutional Review Board at Johns Hopkins Bloomberg School of Public Health (IRB Number-8412), Population Services International, and locally by SIGMA-IRB (CIN No: U74140DL2008PTC182567) and are consistent with international standards for the ethical conduct of research.

## RESULTS

The sociodemographic characteristics of FTPs in pilot and non-pilot cities are similar. For example, 29% of the FTPs in pilot cities and 34% in the non-pilot cities were aged younger than 22 years. Similarly, 80% of the FTPs in pilot cities and 81% in the non-pilot cities were married for less than 5 years, and 13% of FTPs in pilot and non-pilot cities were uneducated (Table 2). A higher proportion of women belonged to the lower wealth tertile in non-pilot areas (47%) compared to pilot areas (27%). The sample distribution was also similar across caste groups and religions. A similar demographic and social status of women between pilot and non-pilot cities indicate that slum populations are homogeneous. However, the difference in household wealth status indicates that there may be socioeconomic heterogeneity within the urban slum population when captured through household-based goods and assets.

**TABLE 2.** Sociodemographic Characteristics Among First-Time Parents Living in Slum Areas Between Pilot and Non-Pilot Cities

** **	**Pilot Cities, %**	**Non-Pilot Cities, %**	**Both, %**
Age of women, years			
15–21	29.4	33.6	30.8
22–24	70.7	66.4	69.2
Marital duration, years			
Less than 5	80.3	81.0	79.7
5 or more	19.7	19.0	20.3
Education in completed years			
None	13.3	13.4	13.8
Less than 6	14.4	10.3	13.1
6–9	23.0	30.0	25.4
12 or more	49.4	46.3	47.7
Wealth tertiles			
Poor	27.3	46.6	33.3
Middle	33.3	33.2	33.3
Rich	39.3	20.2	33.3
Total unweighted number	549	253	802

Source: Output Tracking Survey, 2019.

### Exposure to Family Planning Information Between Pilot and Nonpilot Cities

Exposure to FP information through ASHAs was higher among FTPs in pilot cities (51%) than in non-pilot cities (32%) (Figure). In contrast, exposure to FP information through SDPs was higher among FTPs of non-pilot (53%) than pilot cities (34%). When comparing any exposure to FP-related information, either through ASHAs or through SDPs, 62% of FTPs in the pilot cities were exposed to FP information, whereas in non-pilot cities, this was 66%.

**FIGURE:**
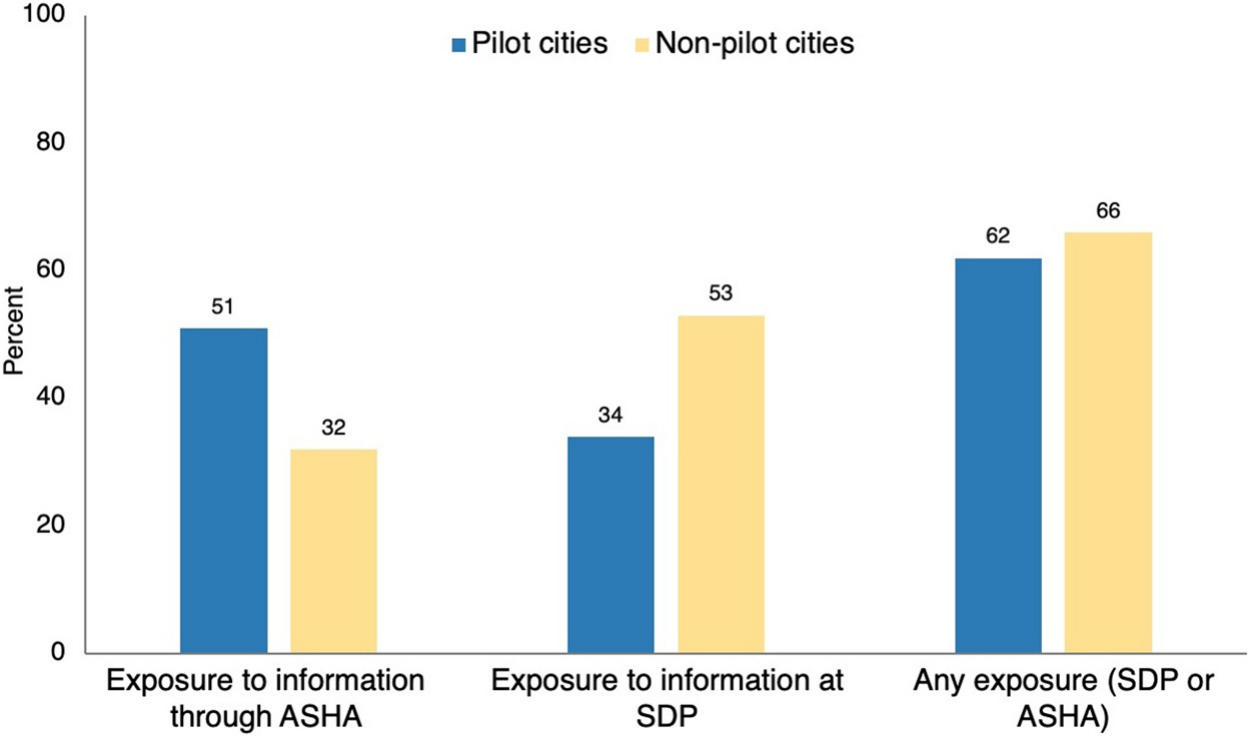
Comparison of FP Information Exposure^a^ Among FTPs Living in Slum Areas in Pilot and Non-Pilot Cities Abbreviations: ASHA, accredited social health activist; FP, family planning; FTP, first-time parents, SDP, service delivery point. ^a^ Information exposure means exposure to government-led general FP programs (non-pilot cities) and exposure to both government-led FP programs and the intervention (pilot cities) in the last 6 months before the survey. Source: Output Tracking Survey, 2019.

Exposure to FP information through ASHAs was higher among FTPs in pilot cities (51%) than non-pilot cities (32%).

### Modern Contraceptive Use Among FTPs

Modern contraceptive use was 39% among FTPs of pilot cities compared to 32% among FTPs in the non-pilot cities (Table 3). This difference is statistically significant *P*-value of <.05. In the case of exposure to FP information either through an ASHA or at an SDP—modern method use was higher among FTPs of pilot cities (41%) than the non-pilot cities (33%), and the difference was statistically significant (*P* value <.05). Modern method use among FTPs who were exposed to FP information through ASHAs was 41% in pilot cities compared to 35% in non-pilot cities; however, the difference was not significant statistically. Similarly, modern contraceptive use among FTPs who were exposed to FP information through visits to SDP was 44% in pilot cities compared to 34% in non-pilot cities. This difference was statistically significant (*P*<.05). Additionally, this is also interesting to observe that in all cases of non-exposure, mCPR was relatively higher, with 35% in pilot cities against 30% in non-pilot cities.

**TABLE 3.** Modern Contraceptive Use Among FTPs in Pilot and Non-Pilot Cities by Exposure to Family Planning Information and Sociodemographic Characteristics

	**Pilot Cities, %**	**Non-Pilot Cities, %**	**Difference, %**
Overall	38.5	31.9	6.6^a^
Exposure to FP information through ASHA			
No	36.4	30.2	6.2
Yes	40.7	35.2	5.5
Exposure to FP information at an SDP			
No	35.6	29.7	5.9
Yes	43.8	33.7	10.1^a^
Any exposure (either through ASHA or SDP)			
No	35.1	30.0	5.1
Yes	40.6	32.8	7.8^a^
Age of women, years			
15–21	33.9	35.8	−1.9
22–24	40.4	29.8	10.6
Marital duration, years			
Less than	41.4	33.5	7.8
5 or more	27.9	24.8	3.1
Education, years			
None	32.6	19.0	13.6
Less than 6	42.8	18.5	24.3
6–9	32.3	30.1	2.2
12 or more	41.9	39.1	2.8
Wealth tertiles			
Poor	26.3	29.1	−2.7
Middle	38.6	35.6	3.0
Rich	48.0	31.4	16.6
Total unweighted number	549	253	

Abbreviations: ASHA, accredited social health activist; FP, family planning; FTP, first-time parent; SDP, service delivery point.

^a^ Significant at 5% using z-test.

Source: Output Tracking Survey, 2019.

### Multivariate Analysis

Binary logistic regression analysis of the interaction between city type and exposure to the FP information indicates that modern contraceptive use was higher among the FTPs of pilot cities who were exposed to FP information compared to the other groups. For example, compared to FTPs in non-pilot cities who were not exposed to FP information through ASHAs, the OR of modern contraceptive use was 1.61 (95% CI=1.04, 2.48) among FTPs in the pilot cities who were exposed to FP information through ASHA (Model I of Table 4). Similarly, in comparison to FTPs in the non-pilot cities who did not visit SDP, the OR of modern method use was 1.84 (95% CI=1.09, 1.31) among FTPs in the pilot cities who visited an SDP for FP. The association was stronger when the FTPs were exposed to FP information through ASHAs as well as SDPs—the odds of contraceptive use was 1.90 (95% CI=1.03, 3.51) among women in the pilot cities who were exposed to FP through ASHAs and SDPs (Model III).

**TABLE 4.** Logistic Regression Analysis^a^ Showing the Interaction Effect of City Type and Program Exposure on Modern Contraceptive Method Use Among FTPs

	**% mCPR**	**Odds Ratio (95% Confidence Interval)**		
		**Model I**	**Model II**	**Model III**
Non-pilot city				
No exposure through ASHA	30.2	Reference		
Exposure through ASHA	35.2	1.40 (0.78, 2.50)		
Pilot city				
No exposure through ASHA	36.4	1.36 (0.88, 2.11)		
Exposure through ASHA	40.7	1.61^b^ (1.04, 2.48)		
Non-pilot city				
No exposure through SDP	29.7		Reference	
Exposure through SDP	33.7		1.33 (0.76, 2.32)	
Pilot City				
No exposure through SDP	35.6		1.40 (0.87, 2.26)	
Exposure through SDP	43.8		1.84^b^ (1.09, 3.11)	
No exposure to ASHA or SDP				
Non-pilot city	30.0			Reference
Pilot city	35.1			1.31 (0.75, 2.31)
Exposure to ASHA only				
Non-pilot city	28.8			0.86 (0.32, 2.34)
Pilot city	36.4			1.40 (0.77, 2.53)
Exposure to SDP only				
Non-pilot city	30.3			1.01 (0.51, 2.03)
Pilot city	40.2			1.53 (0.75, 3.13)
Exposure to ASHA and SDP				
Non-pilot city	38.4			1.74 (0.84, 3.63)
Pilot city	45.8			1.90^b^ (1.03, 3.51)

Abbreviations: ASHA, accredited social health activist; FTP, first-time parent; mCPR, modern contraceptive prevalence rate; SDP, service delivery point.

^a^ Each model was adjusted for age of women, marital duration, education, caste, religion, household wealth, and working status.

^b^ *P*<.05.

Source: Output Tracking Survey, 2019.

This is also interesting to observe the mCPR pattern between pilot and non-pilot cities with similar program exposure through all channels. The odds of contraceptive use was higher among women in pilot cities compared to women in non-pilot cities: 1.61 vs. 1.40 with exposure through ASHAs, 1.84 vs. 1.33 with exposure through SDPs and 1.90 vs. 1.74 with exposure through ASHAs and SDPs, respectively.

## DISCUSSION

This study documented the exposure to the TCIHC-led FP intervention among FTPs in 5 pilot cities of Uttar Pradesh and compared that with FTPs of non-pilot cities. Findings showed higher exposure to FP-related information through ASHAs among FTPs in pilot cities than in non-pilot cities. This might be possible because the program focused on coaching ASHAs to maintain records of age and parity-disaggregated data of eligible women in their catchment areas; meet young and low parity women and inform them about FP services, contraceptive methods, their side effects and management of side effects; and connect women to the nearest health facility to access modern contraceptive methods. This finding suggests that despite having competing priorities in the gamut of maternal and child health, ASHAs in pilot cities prioritize discussing FP-related information with young FTPs. The findings further showed that exposure to FP information from an SDP was greater among FTPs of non-pilot cities than in pilot cities. This might be possible because the greater reach of ASHAs among FTPs in pilot cities provided FP-related information at home. Therefore, the household visits by ASHAs might have minimized the women’s need to visit SDPs to obtain FP information and services. This finding is indicative of ensuring the quality and reach of interpersonal communication during household visits by ASHAs.

In general, the use of modern contraceptive methods was higher among FTPs of pilot cities than in non-pilot cities, irrespective of exposure to FP information. However, among the women who have been exposed to FP information, the use of the modern method was even higher among FTPs in pilot cities than in non-pilot cities, and this was more apparent in the case of exposure to the information with SDPs. These findings indicate that intensive FP programming through ASHA and SDPs can result in higher contraceptive use among young and first parity women in slum areas. This might be possible as community health workers like ASHAs provide an integral link between communities and the public health system, providing access to essential FP services in hard-to-reach areas. Many previous studies have documented that engaging community health workers or conducting interventions through health facilities results in the high use of modern contraceptive methods among young and low-parity women living in marginalized areas.^25^^–^^26^

The use of modern contraceptive methods was higher among FTPs of pilot cities than in non-pilot cities, irrespective of exposure to FP information.

Interestingly, our findings suggest that women who were not exposed to FP information in pilot cities often still had higher modern method use than women in non-pilot who were exposed to the FP information. This may be because reference of information exposure was considered for the 6 months. It may be possible that FTPs in pilot cities were exposed to the information before the 6 months and continued to use methods but were considered to be non-exposed. While in non-pilot cities, women were not exposed and, therefore, not using any method. Another plausible reason could be the influence of the household socioeconomic status measured by wealth index. As only one-fourth of FTPs are poor in pilot cities compared to about half in non-pilot cities, this might have contributed to the higher use of contraceptives among FTPs in pilot cities—even those who were not exposed to the program—compared to their counterparts in non-pilot cities. The analysis also showed that in pilot cities, most of the women from the rich tertile accessed contraceptives from the private sector compared to their counterparts in non-pilot cities (table not shown). These women might have received FP services from the private sector after getting counseling from ASHAs. Previous studies showed that wealth status directly influences the use of modern contraceptives.^20^^–^^21^ This needs to be explored adequately with some future studies.

Our findings further showed that exposure to FP information both through ASHAs and SDPs resulted in a higher and more significant association with modern contraceptive use among FTPs in pilot cities compared to non-pilot cities. Visiting SDPs provides exposure to meet with health service providers and women who have been using contraceptive methods. Facility-based providers, through their exposure to whole-site orientations in the pilot cities, may have been better prepared to counsel FTPs on a larger basket of choice, method suitability, effectiveness, possible side effects, and their management. In contrast, meeting with women who had been adopting a modern method provided an opportunity to learn from experience. All these information exchanges can give better reliance and confidence to FTPs for using a contraceptive method. Previous studies have documented that meeting with facility-based service providers, as well as women who have been already using a method, is related to higher uptake of contraceptive use.^27^^–^^29^

### Limitations

The findings of the study need to be interpreted with several limitations in mind. First and foremost, the pilot cities were in the state of Uttar Pradesh, whereas non-pilot cities were from all 3 TCI-supported states, though mostly from Uttar Pradesh. Therefore, the overall result may be influenced by the state-level variation in proximate determinants of contraceptive use, though to account for this to a certain extent, we adjusted for states in the multivariate analysis. Moreover, the results may have been influenced by the FTPs in non-pilot cities being exposed to government-led generic FP programs with state-level coaching in the TCIHC program. Second, this study used cross-sectional data, which did not allow for drawing causal inferences on many aspects. Third, the lack of pre-intervention survey data limited our ability to compare changes over time in pilot and non-pilot cities. Fourth, while considering the SDPs, we did not include the private sector, and FTPs usually use short-acting reversible contraceptive methods, which they also obtain from the private sector, particularly from shops and pharmacies. Fifth, the short duration of implementation (the survey was conducted after 1 year of implementation) may also have limited the observed results from the survey (i.e., the association of ASHA exposure to contraceptive uptake), especially in the pilot cities. Given a longer duration of implementation with longitudinal research design, we can investigate whether there will be a stronger association seen in pilot cities consistent with the data seen from TCI program management registers where FP client volume increase was higher in pilot cities compared to non-pilot cities.

## CONCLUSIONS

The findings of our study align with some of the previous studies related to behavior change theory and reinforce existing evidence that suggests engaging with government-led community health workers can improve contraceptive use. These findings have important implications for the design and scalability of future programming for young FTPs in India.

Facility-based providers were trained in providing services to adolescents, youth, and FTPs, which may have allowed them to provide more comprehensive, relevant, and responsive consultations and information on FP, resulting in the adoption of modern methods among FTPs. This study further highlights that young FTPs require contraceptives other than sterilization, a need that is often overlooked both by government programs and health providers themselves in India. Greater uptake of modern contraceptives among young women may be achieved when an intentional focus on FTPs is added to the functional FP program. This study’s findings also support the learnings from a previous study (Promoting Change in Reproductive Behavior of Adolescents) that took a more multilevel behavior change approach to increase voluntary contraceptive use for married adolescents and youths.^10^^,^^30^^–^^32^ The findings indicate that such approaches should be accepted and scaled up widely in close collaboration with the government to improve the health and well-being of young FTPs in India facing similar structural and sociocultural barriers. Therefore, the government must continue sponsoring the ESB scheme, promote long-term investments for CHWs, and allocate funding and resources to build the capacities of the health workforce, including ASHAs, to address the issues that prohibit access to a full range of FP services available in the public health system. To mitigate some of the major challenges, policymakers should strengthen the health information system at various levels to promote the visibility and regular review of FP data and establish a culture of prioritization among this group of young married women.

It is important to mention that the association between FP information exposure and contraceptive use among FTPs seen from TCIHC’s FTP initiative in the 5 pilot cities inspired an additional 10 cities in Uttar Pradesh to adopt these programmatic AYSRH best practices and make concerted efforts to reach all FTPs in these cities with FP/RH information and services. Thus, we recommend further assessments on the feasibility and replication of these approaches in other cities with a longer duration of implementation. Future studies are recommended to understand the FP-related needs of FTPs and the accessibility and availability of services and to document the experience of side effects and complications impacting the continuation of FP methods among this population of younger married women.

## Supplementary Material

GHSP-D-22-00170-supplement.pdf

## Notes

**Peer Reviewed**

**First published online:** April 5, 2024.
