# Supplementary material for: Engaging Community Health Workers to Enhance Modern Contraceptive Uptake Among Young First-Time Parents in Five Cities of Uttar Pradesh
Source: Glob Health Sci Pract. 2024 May 21;12(Suppl 2):e2200170. doi: 10.9745/GHSP-D-22-00170 (PMC11111106; doi:10.9745/GHSP-D-22-00170)
Supplement: GHSP-D-22-00170-supplement.pdf [file GHSP-D-22-00170-supplement.pdf]

Supplement to: Sharma MK, Das E, Sahni H, et al. Engaging community health workers to enhance modern contraceptive uptake among young first-time parents in five cities of Uttar Pradesh. *Glob Health Sci Pract*. 2024;12(Suppl 2):e2200170. https://doi.org/10.9745/GHSP-D-22-00170

SUPPLEMENT: FIRST-TIME PARENTS (FTP) PROGRAM DESCRIPTION IN INDIA

Population Services International, India, in partnership with the National Health Mission and with financial support from the William H. Gates Sr. Institute for Population and Reproductive Health at Johns Hopkins Bloomberg School of Public Health has been leading the Challenge Initiative TCI) in India since 2017. TCI, branded as The Challenge Initiative for Healthy Cities (TCIHC) in India, aims to strengthen city-level health service delivery systems to provide family planning (FP) services among the urban poor. TCIHC's service delivery model relies on strengthening urban local governments and community health systems to bring a range of sexual and reproductive health services within the reach of socially and economically vulnerable sections of the urban population.

In Uttar Pradesh (UP), TCIHC initiated its support to strengthen FP services for city-level health systems between October 2017 to August 2018 across 20 selected cities. In August 2018, TCIHC incorporated adolescent youths sexual and reproductive health (AYSRH) interventions into government's urban health service delivery model in 5 of the 20 selected cities of UP, namely Allahabad, Firozabad, Gorakhpur, Saharanpur, and Varanasi. Compared to the remaining cities, the selected 5 pilot cities had a strong National Urban Health Mission platform and functional community health delivery systems such as urban primary health centers with trained staff in place and were therefore considered to be suitable for piloting an initiative focused on addressing the FP/reproductive health (RH) needs of FTPs.

As a first-of-its-kind approach or model in India, TCIHC sought active involvement of local city governments to strategically draw their attention to FTPs under 25 years of age so that this age segment is saturated with information on modern contraception and availability of related services. To achieve a rapid scale-up of service outreach to FTPs, the government with support of initiative "layered" AYSRH activities onto the existing FP/RH service delivery mechanisms in these 5 pilot cities. The package of interventions consisted of strengthening the capacity of urban local governments and health providers at facilities, FP fixed days (FDS) at facilities, coaching and mentoring of community-based frontline health workers, such as ASHAs and advocacy with local government on releasing incentives for ensuring spacing at birth (ESB) schemes

Coaching and Mentoring of ASHAs

ASHAs are the centerpiece of the FTP strategy as they are trained community frontline health workers and potential key influencers that serve as a link between health services and the community in which FTPs reside. Each ASHA typically serves a population of 2000. While ASHA services are voluntary, they can receive performance-based compensation for their work. ASHAs offer community-level FP services by providing condoms, oral contraceptive pills, and emergency contraceptive pills. They counsel eligible couples on contraceptive methods and service delivery points; help women interested in oral contraceptive pills be screened by health providers; and refer women interested in IUDs and injectable

contraceptives to the nearest health facility. ASHAs maintain a list of eligible couples for FP in their catchment area. Apart from these services, ASHAs also support safe motherhood initiatives, immunizations, deliveries, and referrals and escorts to health facilities

ASHAs are required to maintain records and information on general health and FP services that they provided in their respective catchment areas in Urban Health Index Registers (UHIR) also referred to as “ASHA diaries.” A well-maintained UHIR is particularly useful for ASHAs and their supervisors, auxiliary nurse midwives (ANMs), to identify FP users and non-users and ensure they are tailoring their messages for counseling non-users. TCIHC identified several gaps in the maintenance of these registers in urban UP. To begin with, the UHIR was neither printed nor readily available to all ASHAs nor was there any guidance provided on how and when to complete the UHIR and who should review it once it is completed by an ASHA.

In addition, ASHAs did not maintain age and parity disaggregated information on women in their catchment areas in the UHIRs. As a result, FTPs who required referral services or counseling on modern contraception were often missed. To systematize and streamline information on FTPs between the ages of 15 and 24, TCIHC coached and mentored ANMs and Medical Officers to further support ASHAs in completion of UHIR with relevant information on eligible couples including FTPs. TCIHC coached ASHAs to identify potential FTPs, users and non-users of contraception in their respective catchment areas and prioritize household visits and counseling for those in need of FP services as per the prescribed government norms.

To address this gap, TCIHC supported relevant municipal health personnel, including government urban nodal officers and chief medical officers, to estimate the printing needs of UHIRs state-wide and manage printing and distribution to make sure UHIRs were available to all the ASHAs promptly.

As a first step to the ASHA coaching and mentoring effort, two days training and orientation of ASHAs was conducted in 5 pilot cities towards the end of 2018. A total of 994 ASHAs were coached in 45 full day sessions. Further, TCIHC coaches used the ASHA-ANM monthly meeting platform at facilities to coach and mentor ASHAs for FTPs. A total of 12 sessions were planned for ASHAs on topics such as slum mapping and listing, UHIR update for ECs, how to extract FTP data from UHIR, how to counsel FTPs on healthy spacing and connecting with service location, on ESB and follow-up.

ASHAs were coached on the following topics.

- Slum Mapping and Listing: Listing and mapping of urban slums, maintaining a detailed record of households to understand her population in the catchment area.
- UHIR Update: Update the UHIR, focusing on the EC section, helping to prioritize and plan.

- Identify FTPs: Extract FTP data from UHIR to identify users and non-users of contraception and currently pregnant women. Color-code FTPs and prioritize home visits for non-users and follow up with users.
- Reaching and Counseling FTPs: Ensure reaching to 100% FTPs in the community with information on FP and information on facilities where FP services are offered on one-on-one interpersonal communication mode and engaging with gatekeepers like mothers-in-law.
- Connecting FTPs with special FDS: FDS at urban primary health centers celebrate contraception, offering various methods and counseling for FTPs. FDS are organized in a collaborative way once every week at a health facility where trained health personnel (such as medical officer, ANM, staff nurse), equipment, supplies, and commodities are assured on a pre-announced day and time known to the community. Every month, one FDS is dedicated for FTPs. This approach of organizing a fixed day for delivery of FP services helps the government to maximize the use of its limited resources and workforce and provide contraceptive methods and services to large sections of FTPs. Follow-Up: Ensure method retention with follow-ups and encourage healthy spacing through government incentives and schemes like ESB. Under this scheme services of ASHAs to be utilized for counseling newly married couples to ensure spacing of 2 years after marriage and couples with 1 child to have spacing of 3 years after the birth of 1st child.

Critically, completed UHIRs also help ASHAs claim monetary incentives under the government's scheme, ESB, that ASHAs had largely been unaware of and rarely claimed. This served as motivation for ASHAs to reach more FP non-users in their respective catchment areas with comprehensive counseling on the full method mix available to them. The UP government originally introduced the ESB scheme in 2012 intending to improve FP communication and address benefits, myths, and misconceptions about spacing methods among couples. Under the ESB scheme, ASHAs receive monetary remuneration for counseling newly married couples to ensure spacing of 2 years after marriage and couples with 1 child to have a spacing of 3 years after the birth of their first child. While, as mentioned previously, promotion of sterilization methods brought immediate monetary rewards for ASHAs, under ESB ASHAs had to wait for a period of 2 years after the adoption of a spacing method to receive compensation. As a result, these urban ASHAs rarely utilized the ESB scheme.

TCIHC supported government to publicize the ESB scheme and, through meetings, coaching, training and pamphlets, position ESB as an ASHA Investment Plan. ASHAs began to consider UHIR as a long-term investment plan that could motivate them to save for the future when they could easily retrieve information to claim their rightful incentives for promoting spacing methods. This encouraged ASHAs to channel their efforts to promote spacing methods among FTPs and avail the ESB scheme.

Supplement to: Sharma MK, Das E, Sahni H, et al. Engaging community health workers to enhance modern contraceptive uptake among young first-time parents in five cities of Uttar Pradesh. *Glob Health Sci Pract*. 2024;12(Suppl 2):e2200170. https://doi.org/10.9745/GHSP-D-22-00170

At both the facility and community levels, TCIHC supported municipal health staff to overcome provider bias to ensure providers and facility staff were aware of the latest medical guidelines and had accurate knowledge on all the methods available for young FTPs. Below we describe our approach to assess the effectiveness of the TCIHC package of interventions for increasing contraceptive uptake among FTPs in the pilot cities and outline key implications for the replication of this initiative elsewhere in India.
